# Theorising and testing environmental pathways to behaviour change: natural experimental study of the perception and use of new infrastructure to promote walking and cycling in local communities

**DOI:** 10.1136/bmjopen-2015-007593

**Published:** 2015-09-03

**Authors:** Jenna Panter, David Ogilvie

**Affiliations:** Medical Research Council Epidemiology Unit and UKCRC Centre for Diet and Activity Research (CEDAR), University of Cambridge, Cambridge Biomedical Campus, Cambridge, UK

**Keywords:** EPIDEMIOLOGY, PREVENTIVE MEDICINE, PUBLIC HEALTH

## Abstract

**Objective:**

Some studies have assessed the effectiveness of environmental interventions to promote physical activity, but few have examined how such interventions work. We investigated the environmental mechanisms linking an infrastructural intervention with behaviour change.

**Design:**

Natural experimental study.

**Setting:**

Three UK municipalities (Southampton, Cardiff and Kenilworth).

**Participants:**

Adults living within 5 km of new walking and cycling infrastructure.

**Intervention:**

Construction or improvement of walking and cycling routes. Exposure to the intervention was defined in terms of residential proximity.

**Outcome measures:**

Questionnaires at baseline and 2-year follow-up assessed perceptions of the supportiveness of the environment, use of the new infrastructure, and walking and cycling behaviours. Analysis proceeded via factor analysis of perceptions of the physical environment (step 1) and regression analysis to identify plausible pathways involving physical and social environmental mediators and refine the intervention theory (step 2) to a final path analysis to test the model (step 3).

**Results:**

Participants who lived near and used the new routes reported improvements in their perceptions of provision and safety. However, path analysis (step 3, n=967) showed that the effects of the intervention on changes in time spent walking and cycling were largely (90%) explained by a simple causal pathway involving use of the new routes, and other pathways involving changes in environmental cognitions explained only a small proportion of the effect.

**Conclusions:**

Physical improvement of the environment itself was the key to the effectiveness of the intervention, and seeking to change people's perceptions may be of limited value. Studies of how interventions lead to population behaviour change should complement those concerned with estimating their effects in supporting valid causal inference.

**Strengths and limitations of this study**

- In the context of an intervention to change environmental determinants of health, we systematically identified the environmental mediators of changes in walking and cycling in a population-based sample.
- Such evidence for *how* an intervention achieves its effects (causal explanation) can be combined with the evidence for the size of those effects (causal estimation) to provide a stronger basis for causal inference.
- We cannot be certain if changes in mediators led to changes in physical activity, or vice versa, as these were assessed over the same time period. However, most existing research on the mediators of the relationship between physical activity and the environment has been limited to cross-sectional associations, whereas our analysis used longitudinal data from an intervention study.
- We restricted our analysis to participants with complete data on all mediators, which produced a sample for analysis that was somewhat younger and healthier than the main study sample.
- Stronger evidence of mediation might have been found for other unmeasured environmental attributes more closely related to recreational activities, or for other psychological and social constructs such as confidence, intention, self-efficacy or norms.

## introduction

### Physical activity and the environment

Promoting physical activity is a public health priority,^1^ and walking and cycling are potential targets for intervention strategies because they are relatively easy to integrate into daily life and may confer substantial individual health benefits^2^ and wider social and environmental co-benefits.^3^ ^4^ However, efforts to encourage walking and cycling at the population level have met with modest success to date.^5–7^ It is argued that changing the environment may be required to produce broader and more sustained effects, but this is mostly based on evidence from cross-sectional observational studies. These suggest that factors such as distance to destinations, density and land use mix may be important influences on walking and cycling,^8^ ^9^ but there are few longitudinal studies examining the environmental determinants of behaviour change or evaluating the impact of environmental changes.^5–11^ While these latter types of study are gradually shifting the focus of research from correlation towards causation, they sometimes report null associations for environmental attributes found to be significant in cross-sectional studies.^11^ Even if well designed studies have provided a relatively unbiased estimate of the effect size for an environmental intervention (causal estimation), some authors argue that valid causal inference in public health also depends on showing *how* an intervention brings about the outcomes attributed to it (causal explanation).^12^ ^13^

### In search of causal explanation for environmental interventions

Socioecological models postulate that intrapersonal, interpersonal and community-level environmental factors are important influences on health behaviours, and these have been shown to be important for physical activity.^14^ However, these models generally provide a broad framework indicating the existence of such influences at multiple levels, rather than considering *specifically* how behaviour is postulated to change in response to environmental changes. Understanding such mechanisms could be expected to clarify the significance and role of specific factors along the putative causal pathway linking environmental change to physical activity behaviour change,^12^ ^13^ ^15^ but few studies have attempted to do this.^11^ This may reflect the fact that the causal pathways for public health interventions can be long and complex.^13^ Nevertheless, investigating how changes to the environment are perceived and acted on could provide greater understanding of how interventions work and thereby inform the design and targeting of future interventions.^13^ ^15^

### The iConnect study

Connect2 is a programme of projects to promote walking and cycling at 79 sites around the UK. Each comprises a core engineering project such as a bridge over a busy road, railway or river, which together with the development or improvement of feeder routes was intended to make it easier for pedestrians and cyclists to reach destinations in their local area (http://www.lotterygoodcauses.org.uk/project/sustrans-connect2). The iConnect study began with the development of a general theoretical framework and a preliminary intervention model that was used to guide data collection and analysis.^16^ Briefly, the model postulated that a Connect2 project may alter the physical accessibility of local destinations and other potentially relevant characteristics of the environment. It was always intended that this preliminary intervention model would be tested and refined in longitudinal analysis.^16^ The main outcome evaluation has shown positive effects of the intervention on walking, cycling and overall physical activity after 2 years,^17^ and qualitative interviews have highlighted the potential importance of visibility of the new infrastructure in fostering behaviour change in local people.^18^ In this paper, we build on these findings by investigating the ‘environmental’ mechanisms linking the intervention with behaviour change. We did not set out to test all the potential causal mechanisms for behaviour change in this context, such as those involving psychological constructs such as confidence, intention or self-efficacy. Instead we have focused on that part of the causal pathway most proximally related to the intervention, which relates to perceptions of changes in the supportiveness of the environment for walking and cycling, such as the convenience and safety of routes, and use of the new infrastructure. We systematically describe and test a series of hypothesised mediating processes, seeking to identify not only which mediators are important but also their most plausible causal ordering. We then use the findings to refine the overall intervention model and subsequently to assess the relative contributions of the different pathways to behaviour change.

## Methods

### Intervention, settings and data collection procedures

A more detailed description of the intervention, settings and data collection procedures is available elsewhere.^19^ Briefly, three Connect2 projects in Cardiff, Kenilworth (Warwickshire) and Southampton were purposively selected as case study sites according to criteria including implementation timetable, likelihood of measurable population impact and heterogeneity of overall mix of sites, including the composition of the local population and the topographical context.^16^ ^18^ ^19^ In Cardiff, pedestrians and cyclists travelling between the city centre and the suburbs across Cardiff Bay had to share space with motor vehicles on a busy road, and the centrepiece of the Connect2 project was a new 140 m long, 4 m wide traffic-free bridge with integral lighting. In Kenilworth, a new traffic-free bridge was built across a busy trunk road to link the town to a rural greenway, and in Southampton, a new 400 m boardwalk was built along the shore of the tidal River Itchen, replacing an informal footpath which was impassable at high tide. Each project included improvements to feeder routes which linked the new infrastructure with existing route networks.

Questionnaires were posted to 22 500 adults aged 18 and over who were listed on the edited electoral register as living within 5 km by road of the core Connect2 project at any of the three sites in April 2010. Information on demographic and socioeconomic characteristics, travel and physical activity behaviours, and perceptions of the environment were collected, and additional questions were asked at follow-up to assess use of the Connect2 project. The questionnaire is published in full elsewhere.^19^ In total, 3516 individuals returned questionnaires at baseline, of whom 1510 (43%) also returned questionnaires at 2-year follow-up in April 2012 after the opening of the new infrastructure. All participants provided written informed consent.

### Measures

As the main outcome evaluation showed that residential proximity to the new routes predicted increases in weekly time spent walking and cycling (the primary outcome),^17^ we used the same measures of intervention exposure and outcome in this analysis.

#### Exposure

Those living closer to the Connect2 projects were deemed to be more highly exposed to the intervention than those living further away. Proximity to Connect2 was assessed using the shortest distance between each participant's home address and the nearest access point to the Connect2 project (including feeder routes) using an enhanced road network which included traffic-free and informal paths.^19^

#### Outcome

Walking and cycling for transport were assessed using a 7-day recall instrument covering journeys made for five purposes: for commuting, on business, for study, for shopping and personal business, and for social activities.^19^ Participants reported the total time spent walking and cycling for travel for each purpose, and these were summed across all purposes for each mode of travel. Recreational physical activity was measured using an adapted version of the short form of the International Physical Activity Questionnaire in which participants reported the total time spent walking for recreation and cycling for recreation in the past week.^20^ Total weekly time spent walking and cycling was derived by summing the times spent walking and cycling for transport and for recreation, and change scores were computed as the time reported at follow-up minus the time reported at baseline.

#### Mediators

We hypothesised that the effects of Connect2 on overall walking and cycling might come about as a result of participants’ awareness of improvements in the physical and social environmental conditions for those behaviours and their use of the new routes, which we investigated as potential environmental mediators. At both time points, participants were asked to report their agreement with seven items referring specifically to the physical environment traversed by the Connect2 project, using a five-point Likert scale from strongly disagree (−2) to strongly agree (+2) (table 1). Four additional items asked about the visibility of walking for travel, walking for recreation, cycling for travel and cycling for recreation in terms of whether participants saw people engaging in these behaviours ‘in my neighbourhood’. Change scores for each of the physical environmental items were computed as the difference between the baseline and follow-up measures, while change in the visibility of walking and cycling was summarised using the mean of the corresponding change scores for the four individual items to match the outcome of total weekly time spent walking and cycling. At follow-up, participants were also asked if they had walked or cycled on the Connect2 project (yes/no).

**Table 1:** Items assessing (changes in) the perceived physical and social environment and rotated factor loadings

Description	Item	Factor 1 *Change in infrastructure*	Factor 2 *Change in safety*
Perceived physical environment			
Safety for walking	Walking is unsafe because of the traffic	0.276	0.809
Safety for cycling	Cycling is unsafe because of the traffic	0.243	0.804
Pavements for walking	There are pavements suitable for walking	0.732	0.221
Special lanes for cycling	There are special lanes, routes or paths for cycling	0.688	0.280
Pleasant	The routes are pleasant for walking or cycling	0.706	0.203
Low crime	The level of crime or antisocial behaviour means walking or cycling is unsafe	−0.128	0.678
Lighting	The routes for walking and cycling are generally well lit at night	0.695	0.032
Perceived social environment			
Visibility of cycling for transport	I see people in my neighbourhood cycling for travel	NA	NA
Visibility of walking for transport	I see people in my neighbourhood walking for travel	NA	NA
Visibility of cycling for recreation	I see people in my neighbourhood cycling for recreation	NA	NA
Visibility of walking for recreation	I see people in my neighbourhood walking for recreation	NA	NA

NA: not applicable as this variable was not used in factor analysis. Factor analysis was based on 1211 participants for whom change scores for all relevant items were available.

#### Covariates

All demographic (sex, age, ethnicity and presence of any child under 16 in the household), socioeconomic (highest educational level, annual household income and employment status) and health variables (height, weight, general health, and presence of long-term illness or disability limiting daily activities) were self-reported at baseline. Height and weight were used to compute body mass index and assign participants to one of three categories of weight status based on internationally recognised cut-offs.^21^

### Analysis

Our analysis was divided into three steps. We first explored the factor structure of the items assessing perceptions of the physical environment, to identify whether groups of items were related and changed in similar ways (step 1: see below). This reflected the fact that the Connect2 projects aimed to improve the environment for walking and cycling more generally, rather than targeting single aspects such as safety or pleasantness. We then identified candidate mediators and their most plausible conceptual ordering by systematically exploring the associations between the environmental perception measures (factor scores for the physical environmental items, and the mean change score for the visibility items), proximity to and use of Connect2, and change in time spent walking and cycling (step 2). Having thereby refined our intervention theory, we then used path analysis—a confirmatory analysis technique—to formally test the model and estimate the magnitude and significance of the hypothesised causal relationships between the sets of variables (step 3).^22^ All analyses were restricted to participants who had not moved home during the study and whose total reported physical activity had not changed by >900 min/week, which may have come about as a result of misreporting (eg, misreporting 15 min as 15 h). Steps 1 and 2 were conducted using STATA, and step 3 using Mplus.

#### Step 1: Factor analysis of changes in perceptions of the physical environment

A principal components analysis was conducted on the items assessing perceptions of the physical environment at baseline and at follow-up, as well as on the change scores. Factors with an eigenvalue less than one were dropped; factor loadings were rotated using varimax (orthogonal) rotation and factors were scored by the method suggested by Bartlett,^23^ creating scores for each factor weighted according to the item loadings.^24^ These analyses were further restricted to participants who had completed all the physical environmental perception items at both time points.

#### Step 2: Identification of mediators and refinement of intervention theory

We systematically tested the associations (1) between proximity to Connect2 and the hypothesised mediators (changes in the environmental perception measures and use of Connect2); (2) between these hypothesised mediators and change in walking and cycling; and (3) between the various mediators. We fitted separate linear or logistic regression models as appropriate for all the associations tested. These were adjusted for total weekly time spent walking and cycling at baseline and all the demographic, socioeconomic and health characteristics listed above, but were not adjusted for the other mediators. The objective was not to isolate statistically significant single associations, but to identify plausible links in a causal pathway to be carried forward to the next stage of analysis, as advocated by Victora *et al*.^13^ We therefore applied a generous criterion of p<0.25 to identify ‘plausible’ associations at this stage. However, because the aim of the analysis was to elucidate mechanisms for an *intervention* that had already been shown to be positively associated with the behaviour change outcomes, we carried forward only those mediators that were directly associated with both the exposure and either the outcome or another mediator, and for which all the observed associations were in the expected (ie, positive) direction.

#### Step 3: Testing the intervention model

The resulting model was tested using path analysis, in other words using a structural equation model with no latent variables. This approach allows sets of relationships between variables to be modelled simultaneously, using linear or logistic regression as appropriate according to the form of the dependent variables and with the mediating variables being treated as both dependent and independent variables.^25^ It is a confirmatory form of analysis in which a model depicting unidirectional causal effects of one variable on another is tested with no possibility of incorporating feedback loops.^26^ We adopted a complete case approach, restricting these analyses to participants who had provided data on exposure, outcome, and all mediators and covariates, and used maximum likelihood estimation with 1000 iterations.

#### Stratified analyses

We further hypothesised that different mechanisms of behaviour change might have operated in people with different levels of walking and cycling prior to the intervention. We therefore divided the sample at the median total time spent walking and cycling at baseline (190 min/week) and repeated steps 2 and 3 in the low-active and high-active subgroups. Because there remained significant variation in baseline activity within each subgroup, we also adjusted for time spent walking and cycling at baseline in these models.

## Results

### Sample characteristics

Of the 1510 participants who returned survey data at baseline and follow-up, 1465 met the inclusion criteria for the main outcome evaluation (had neither moved home nor reported a large change in physical activity) and 1211 provided information sufficient for the factor analysis in step 1 in this analysis. The sample size for each regression model in step 2 ranged from 969 to 1139 according to the completeness of reporting of the various mediators. In total, 967 participants provided complete data on exposure, outcome, and all mediators and covariates, and comprised the sample for the analysis in step 3. Compared with the sample of 1465 used for the main outcome evaluation,^17^ our final subsample was slightly younger on average and included a higher proportion of men (table 2). Participants in this final subsample were also more likely to be educated to tertiary level, to have access to a car and to a bicycle and to have a child in their household, and less likely to report having a long-term health condition (all p<0.001). However, our subsample was not significantly different from the main sample in terms of ethnicity, weight status or time spent walking and cycling at baseline.

**Table 2:** Characteristics of the sample

Variable	Category	Participants providing data on exposure and outcome (n=1465), % (N)	Participants providing data on exposure, outcome and all mediators and covariates (n=967), % (N)
Site	Cardiff	32.3 (473)	33.6 (325)
	Kenilworth	39.9 (584)	40.7 (394)
	Southampton	27.9 (408)	25.7 (248)
Residential proximity to intervention (km)	≥4	9.6 (141)	9.7 (93)
	3–3.99	7.0 (103)	6.9 (66)
	2–2.99	15.2 (222)	15.2 (147)
	1–1.99	32.4 (474)	31.6 (306)
	<1	35.8 (525)	36.6 (355)
Sex	Female	56.7 (831)	51.9 (502)
	Male	43.3 (634)	48.1 (465)
Age (years) at baseline	18–34	9.7 (141)	11.0 (107)
	35–49	19.9 (291)	24.1 (233)
	50–64	35.5 (519)	38.5 (372)
	65–89	34.9 (510)	26.4 (255)
Ethnicity	Caucasian	96.9 (1417)	97.2 (940)
	Non-Caucasian	3.1 (45)	2.8 (27)
Any child under 16 in household	No	84.4 (1236)	81.1 (784)
	Yes	15.6 (229)	18.9 (183)
Highest educational level	Tertiary or higher	39.5 (576)	45.9 (444)
	Secondary school	32.8 (479)	32.9 (318)
	Lower than secondary	27.7 (405)	21.2 (205)
Annual household income	>£40 000	32.1 (439)	36.6 (355)
	£20 001–£40 000	33.7 (461)	35.0 (337)
	≤£20 000	34.3 (469)	28.4 (275)
Employment status	Working	49.2 (720)	56.7 (548)
	Student	1.6 (24)	1.4 (14)
	Retired	40.3 (589)	33.2 (321)
	Other	8.9 (130)	8.7 (84)
Any car in household	No	13.9 (203)	10.0 (97)
	Yes	86.1 (125)	90.0 (870)
Any adult bicycle in household	No	44.6 (603)	39.5 (382)
	Yes	55.4 (748)	60.5 (585)
Weight status	Normal/underweight	49.0 (683)	48.4 (468)
	Overweight	37.0 (515)	37.6 (363)
	Obese	14.0 (195)	14.0 (136)
General health	Excellent/good	78.5 (113)	81.6 (789)
	Fair/poor	21.5 (312)	18.4 (178)
Long-term illness or disability that limits daily activities	No	74.0 (102)	78.1 (756)
	Yes	26.0 (359)	21.9 (211)
Time spent walking and cycling in past week (min)	None	15.6 (229)	14.0 (135)
	1–149	25.7 (376)	27.2 (263)
	150–299	23.5 (344)	23.6 (229)
	300–449	14.4 (211)	14.2 (138)
	≥450	20.8 (305)	20.9 (202)

#### Step 1: Factor analysis of changes in perceptions of the physical environment

The results of the factor analyses of the baseline and follow-up values were similar to those of the factor analysis of the change scores (see online supplementary file 1). We therefore chose to use the factors and factor scores derived from the change scores. We identified two meaningful factors, which we described as representing perceived changes in infrastructure (eigenvalue: 2.9) and perceived changes in safety (eigenvalue: 1.2; table 1). These factors explained 58% of the variance in the change scores for the physical environmental perception items.

#### Step 2: Identification of mediators and refinement of intervention theory

##### Whole sample

Table 3 summarises the associations between the putative mediators, proximity to Connect2 and change in time spent walking and cycling. As reported elsewhere,^17^ ^27^ proximity to Connect2 was associated with use (OR=1.85, p<0.001; table 3A) and use of Connect2 was associated with change in time spent walking and cycling (β=31.16, p=0.06; table 3A). Proximity to Connect2 was associated with perceived changes in infrastructure and visibility (both β=0.05, p≤0.03) and safety (β=0.03, p=0.18; table 3C). Although all of these also met the criteria for a plausible association with use of Connect2 (1.23<OR<1.33, all p<0.008; table 3D), only a perceived change in safety was directly associated with change in time spent walking and cycling (β=9.19, p=0.22; table 3E). The association between perceived changes in infrastructure and visibility also met the criteria for inclusion (β=0.06, p=0.04), while those between perceived changes in safety and infrastructure or visibility did not (table 3F).

**Table 3:** Associations between potential mediators, proximity to intervention and change in walking and cycling

**(A) Associations between proximity to and use of intervention**		
Independent variable: residential proximity to intervention (km)		
**Dependent variable**	**OR (95% CI)**	**p Value**
Use of intervention (yes/no)	1.85 (1.61 to 2.11)	0.001

**(B) Associations between use of intervention and change in walking and cycling**		
Independent variable: use of intervention (yes/no)		
**Dependent variable**	**β (95% CI)**	**p Value**
Change in time spent walking and cycling (min/week)	31.16 (−1.72 to 64.05)	0.063

**(C) Associations between proximity to intervention and perceived environmental changes**		
Independent variable: residential proximity to intervention (km)		
**Dependent variable**	**β (95% CI)**	**p Value**
Change in infrastructure	0.05 (0.01 to 0.10)	0.030
Change in safety	0.03 (−0.02 to 0.08)	0.182
Change in visibility	0.05 (0.01 to 0.10)	0.013

**(D) Associations between perceived environmental changes and use of intervention**		
Dependent variable: use of intervention (yes/no)		
**Independent variable**	**OR (95% CI)**	**p Value**
Change in infrastructure	1.23 (1.06 to 1.44)	0.008
Change in safety	1.31 (1.13 to 1.54)	0.001
Change in visibility	1.33 (1.15 to 1.55)	0.001

**(E) Associations between perceived environmental changes and change in walking and cycling**		
Dependent variable: change in time spent walking and cycling (min/week)		
**Independent variable**	**β (95% CI)**	**p Value**
Change in infrastructure	−2.51 (−17.16 to 12.13)	0.736
Change in safety	9.19 (−5.36 to 23.74)	0.215
Change in visibility	−6.21 (−20.62 to 8.19)	0.398

**(F) Associations between perceived environmental changes**		
Dependent variable: change in visibility		
**Independent variable**	**β (95% CI)**	**p Value**
Change in infrastructure	0.06 (0.00 to 0.12)	0.039
Change in safety	0.03 (−0.03 to 0.09)	0.328
Dependent variable: change in safety		
**Independent variable**	**β (95% CI)**	**p Value**
Change in infrastructure	−0.03 (−0.10 to 0.03)	0.215

Linear or logistic regression models as appropriate adjusted for time spent walking and cycling at baseline and the demographic, socioeconomic and health characteristics shown in table 2. Proximity was modelled as the negative of the distance between home and the nearest access point to the ‘greater Connect2 project’ including feeder routes. Each row represents a separate model which was not adjusted for the other mediators.

**Figure 1:**
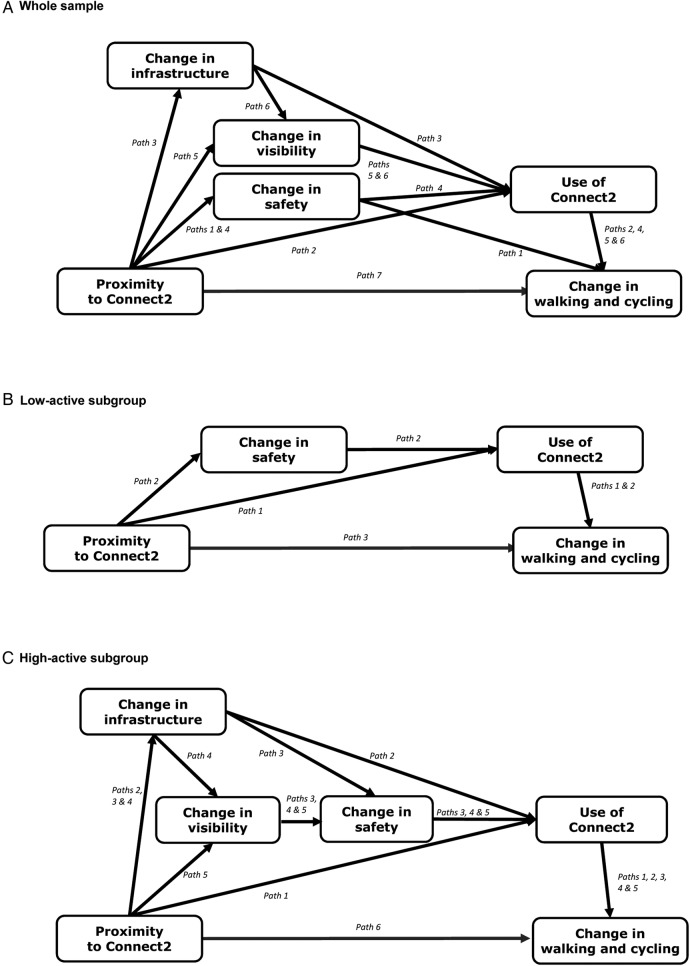
Path models fitted in Mplus.

Based on these results, a path model was developed to capture the most plausible theory of change linking proximity to the intervention with change in time spent walking and cycling (figure 1A). Perceived changes in infrastructure, safety and visibility were all associated with proximity, and because these were hypothesised to change as a direct and proximate result of the intervention they were placed directly after proximity in the model. All three perceived changes were also associated with use of the intervention, and we assumed that the more plausible causal ordering was that the changes in the perceived supportiveness of the environment may have led to use of the new infrastructure. Use was also associated with proximity and with change in time spent walking and cycling, so we included an additional indirect path between exposure and outcome via use only. Only one of the inter-relationships between the perceived environmental changes—that between infrastructure and visibility—was identified as plausible, and we assumed that perceived improvements in infrastructure were more likely to reflect a direct and proximate effect of the *physical* intervention and may therefore have preceded the perceived change in the visibility of walking and cycling. Given the lack of clear theory or evidence in relation to the causal ordering of some of these mediators, however, we developed two alternative models that were also consistent with the associations observed in step 2: one in which the perceived change in visibility preceded the perceived change in infrastructure (see online supplementary file 2; alternative 1), and one in which the perceived change in safety followed use of the infrastructure (see online supplementary file 2; alternative file 2).

##### Low-active subgroup

In the low-active subgroup, proximity to Connect2 was associated with use (OR=2.05, p=0.001), and use of the infrastructure was associated with change in time spent walking and cycling (β=62.96, p<0.001; online supplementary additional file 3a, 3b). Proximity was associated with perceived changes in safety (β=0.08, p=0.03) and infrastructure (β=0.05, p=0.15), but the association with change in visibility did not meet the criteria for inclusion (see online supplementary additional file 3c). Perceived changes in safety and visibility, but not in infrastructure, met the criteria for a plausible association with use of Connect2 (1.14<OR<1.42, p<0.25; online supplementary additional file 3d). None of the associations between the putative mediators and change in time spent walking and cycling met the criteria for inclusion, nor did those between the various perceived environmental changes (see online supplementary file 3e, 3f). While perceived changes in infrastructure and visibility therefore met the criteria for inclusion in the model based on single associations (with proximity and use, respectively), they could not be linked on a pathway and were therefore deemed not to be plausibly causally related to the effects of the intervention in this subsample. Perceived change in safety and use of the infrastructure were therefore the only mediators included in the model for this subgroup (figure 1B).

##### High-active subgroup

Similarly, in the high-active subgroup, proximity to Connect2 was associated with use of the infrastructure (OR=1.79, p=0.001), and use was associated with change in time spent walking and cycling (β=83.97, p<0.001; see online supplementary file 4a, 4b). Proximity was associated with perceived changes in visibility and infrastructure (0.06<β<0.10, both p<0.08), but the association with perceived change in safety did not meet the criteria for inclusion (see online supplementary file 4c). All three perceived environmental changes met the criteria for a plausible direct association with use of Connect2 (1.21<OR<1.58, p<0.09), but not with change in time spent walking and cycling (see online supplementary additional file 4d, 4e). The association between perceived changes in infrastructure and visibility also met the criteria for inclusion (β=0.11, p=0.02; online supplementary file 4f). Based on these results, we developed the path model shown in figure 1C.

#### Step 3: Testing the intervention model

The model shown in figure 1A was fitted in path analysis for the whole sample (table 4). The effect of proximity to the intervention on change in time spent walking and cycling was almost entirely explained by an indirect path via use of the infrastructure (path 2, 90%), while the remaining indirect paths that included perceived changes in infrastructure, safety or visibility together explained only 8% of the effect. Path analysis of the alternative models incorporating different causal ordering of the mediators gave very similar results (see online supplementary file 2), as did path analysis of the models for the low-active and high-active subgroups (see online supplementary file 5).

**Table 4:** **C**ontributions of different pathways to behaviour change

Path	β (95% CI)	Per cent of effect explained
Indirect via safety only (path 1)	0.21 (−0.68 to 1.09)	0.4
Indirect via use only (path 2)	43.13 (22.09 to 64.17)	89.9
Indirect via infrastructure and use (path 3)	1.33 (0.03 to 2.63)	2.8
Indirect via safety and use (path 4)	1.38 (−0.04 to 2.81)	2.9
Indirect via visibility and use (path 5)	0.76 (−0.14 to 1.65)	1.6
Indirect via infrastructure, visibility and use (path 6)	0.09 (−0.03 to 0.20)	0.2
Direct (path 7)	1.09 (−9.63 to 11.81)	2.2
Total (sum of paths 1–7)	47.99 (26.32 to 69.66)	100

Model shown in figure 1A fitted using path analysis in Mplus, adjusted for time spent walking and cycling at baseline and the demographic, socioeconomic and health characteristics shown in table 2.

## Discussion

### Principal findings

In this study, we have refined and tested key components of a theoretical model linking the provision of new walking and cycling routes with changes in walking and cycling behaviour in local communities. In doing so, we have made both methodological and substantive contributions to the challenge of evaluating and understanding the effects of interventions to change the environmental determinants of health, which are understood to work through long and potentially complex causal pathways.^13^ Having previously developed a provisional intervention model, we systematically identified the most plausible mediators, associations and causal ordering; refined the model; and then formally tested the model using path analysis. We found that exposure to the intervention was associated with changes in the perceived supportiveness of the physical and social environments for walking and cycling, even after adjustment for baseline levels of those behaviours and other potential confounders. This suggests that the intervention was at least somewhat successful in changing those aspects of the environment. However, path analysis showed that the effects of the intervention on changes in walking and cycling were largely explained only by use of the new infrastructure, and that other explanatory pathways involving changes in cognitions relating to the environment explained only a small proportion of the effect. This overall finding was replicated in separate analyses restricted to participants with lower or higher levels of activity at baseline, although there were differences in the specific patterns of associations observed.

### Strengths and limitations

In the context of an intervention to change environmental determinants of health, we have systematically identified the most important environmental mediators and their most plausible causal ordering, and tested and compared a series of mediating pathways, in order to improve our theory of how such interventions may work. Our study was conducted as a natural experiment using general population samples drawn from three contrasting communities, which confers a degree of external validity that may be lacking from some behavioural research conducted in less natural settings. A further strength lies in the specificity of the measures of perceptions of the physical environment, which were both specific to the area traversed by the intervention and hypothesised to change as a direct result of the intervention. Our approach to analysis was underpinned by a specific preliminary theoretical model for the intervention,^16^ and the pathways tested were consistent with the principles outlined in more general behavioural frameworks such as the Environmental Research framework for weight Gain prevention (EnRG).^28^ While the testing and refinement of theory in this way is commonly applied in the analysis of qualitative data,^29^ it is less commonly (or explicitly) applied in the statistical analysis of quantitative data in public health research. This study therefore offers a methodological contribution to the challenge of evaluating and understanding complex public health interventions, an area in which both the theory of behaviour change and the methods for evaluation remain underdeveloped.^29^ Partly for this reason, we used generous statistical criteria to identifying plausible pathways for further testing and we also tested some alternative model configurations, which showed that our assumptions about the causal ordering of mediators made little difference to the relative importance of the main pathways identified. We have tried to document our methods as clearly as possible in the hope that other researchers will adapt and refine our methods, investigate the replicability of our findings in other populations and settings, and explore the wider applicability of this approach in public health research.

Nevertheless, this study had several important limitations. First, we restricted our analysis to participants with complete data on all mediators, which produced a sample for analysis that was somewhat younger and healthier than the main study sample.^17^ This, together with the low initial response rate, means that our sample cannot be assumed to be representative of the local resident populations. Second, although our measures of perceptions of the physical environment were highly specific, we used more composite measures of perceptions of the social environment and of the behavioural outcomes, to ensure comparability with the main outcome evaluation and because the largest intervention effect was observed for the composite outcome of overall time spent walking and cycling.^17^ We acknowledge the need for further investigation of more specific exposure–outcome relationships which may shed more light on how changes in specific behavioural outcomes come about.^30^ Third, because changes in putative mediators and changes in behaviour were assessed over the same time period, we cannot be certain if changes in mediators led to changes in physical activity or vice versa. On the contrary, whereas most existing research on the mediators of the relationship between physical activity and the environment has explored only cross-sectional associations, which provide little basis for causal inference,^31–33^ a key strength of our analysis is that it used longitudinal data from an intervention study in which environmental changes were known to have been introduced and could reasonably be assumed to have causally preceded the changes observed.^11^

### Understanding intervention mechanisms to strengthen the basis for causal inference

Our investigation not only provides greater understanding of the causal explanation of how behaviour change comes about as a consequence of an environmental intervention, but also provides a stronger basis for causal attribution. This was a natural experimental study in which participants were not randomised to allocation status, but were exposed to the intervention to a greater or lesser extent according to the proximity of their home to the new infrastructure. In studies of this kind, we can never be entirely sure that the analysis of the main effect is free from residual confounding by unobserved variables, which can neither be controlled for in analysis nor assumed to be balanced between groups as in a randomised controlled trial.^34^ ^35^ However, we have demonstrated a plausible, logical and parsimonious pathway linking geographical exposure to the intervention via individual use of the intervention to individual changes in walking and cycling behaviour, and we have shown that this mechanism explains the large majority of the effect of the intervention. This evidence for *how* an intervention achieves its effects (causal explanation) can be combined with the evidence for the size of those effects (causal estimation) reported elsewhere^17^ to provide a stronger basis for valid causal inference.^13^

### Identifying modifiable perceptions of the physical and social environment

The rationale for selecting intervention sites for the Connect2 programme was to improve provision for local walking and cycling journeys in places where existing provision was poor. For example, the project in Cardiff involved providing a new traffic-free river crossing as an alternative to sharing space with motor vehicles on a busy road bridge or making a long detour,^19^ factors which qualitative research with local informants identified as barriers to walking or cycling.^18^ In our analysis, proximity to and use of the intervention both showed significant associations with perceived changes in infrastructure and safety for walking and cycling and with the perceived visibility of those behaviours in the neighbourhood. This provides some evidence that the Connect2 programme was successful in influencing these characteristics of the environment, and that these changes may have contributed to people taking up the opportunity to use the new infrastructure. Restricting the analysis to participants with a higher level of activity at baseline revealed a similar pattern of associations to that observed in the whole sample, whereas in the low-active subgroup, a perceived change in safety was the only environmental mediator found to be associated with both exposure and use. Consistent with findings from some cross-sectional and longitudinal studies,^36^ this suggests that improving safety—reflected in this study by survey questions about safety from crime or antisocial behaviour, as well as safety from traffic—may be particularly important in promoting the use of walking and cycling routes among those with the most capacity to benefit from an increase in physical activity.

### The role of behaviour-specific cognitions in behaviour change

Despite the fact that perceived changes in the physical and social environment were reported by people living in the areas served by the Connect2 projects and associated with use of the new routes, we found that pathways between intervention exposure and behaviour change involving these perceived changes explained a very small percentage of the intervention effect, 90% of which was accounted for by use of the intervention alone. This may appear a slightly unexpected finding, given the body of cross-sectional evidence suggesting a relationship between physical activity behaviours and the perceived supportiveness of the environment.^8^ ^9^

Perceived environmental changes were only weakly associated with changes in time spent walking and cycling, suggesting that they played a relatively small part in determining overall behaviour change in the sample. Importantly, the largest contributor to the increase in overall time spent walking and cycling was an increase in recreational walking,^17^ whereas at baseline, perceptions of the environment were generally more strongly associated with walking or cycling for transport than with walking or cycling for recreation.^37^ The latter finding is consistent with existing literature in which attributes of the environment have been found to have mixed patterns of associations with walking and cycling, and with recreational and transport activities.^8^ ^9^ It is therefore possible that stronger evidence of mediation might have been found for other unmeasured environmental attributes more closely related to recreational activities (or indeed for other psychological constructs such as confidence, intention or self-efficacy, which were not the focus of this study).

An alternative interpretation of the weak evidence for the mediating role of behaviour-specific cognitions in this study is that it supports the notion of more automatic, unconscious processes linking environmental change with behaviour change. Behavioural scientists have described how behaviour may be determined by a more reflective, goal-orientated system on the one hand or by a more automatic, affective system on the other,^38^ and Kremers *et al*^28^ have specifically referred to both ‘mediated’ and ‘unmediated’ pathways in the context of the influence of the environment on energy-related behaviours. Our findings could be regarded as consistent with, although certainly not proof of, the hypothesis that physical activity behaviour change can be promoted by altering relevant environmental cues—sometimes referred to as changing choice architecture^39^ or ‘nudging’^40^—without explicitly encouraging the target behaviours or directly addressing people's perceptions and other cognitions relating to them.^41^ Indeed, the fact that behaviour change in this study was strongly associated with proximity to and use of the infrastructure, but only weakly associated with people's perceptions of how the environment had changed, suggests that the physical improvement of the environment itself—rather than the modification of people's perceptions of their environment—was the key to the effectiveness of the intervention.

### Implications for future research

As many authors have pointed out, few studies have evaluated the effects of environmental approaches to changing population physical activity behaviour, and even fewer have gone beyond estimating their effects to investigate the mechanisms underlying the (in)effectiveness of interventions.^5^ ^10^ ^11^ ^42^ ^43^ Complementary evidence of effects and mechanisms will help strengthen the case for causal inference, particularly in a field in which randomised controlled trials are rarely feasible.^12^ ^13^ More work is required to refine the hypotheses about how specific interventions may work and to generate improved measures to reflect the proposed mechanisms. The former might include investigating the social (collective) mechanisms of behaviour change and their interaction with individual factors. For example, it is unknown whether the impact of environmental change is more or less important for those with different attitudes to physical activity, and some authors have suggested the existence of synergistic or competitive mechanisms.^44^ The latter might include developing objective measures of the nature, extent, timing and quality of environmental change,^45^ as well as detailed individual-level measures of the ‘dose’ of intervention received—such as exposure to and use of new environments—and of how interventions are received and interpreted. Improved measures of this kind will enable the hypothesised pathways to behaviour change to be tested—and preferably reported as transparently as possible, as recommended by the authors of a recent review^46^—in order to identify the most promising strategies for future interventions to change the environmental determinants of health.

## Conclusions

Local residents’ perceptions of the supportiveness of the physical and social environment for walking and cycling were changed after the construction of new infrastructure in their communities. However, the effect of the intervention on overall walking and cycling was largely explained by a simple causal pathway involving use of the new routes, and other explanatory pathways involving changes in cognitions relating to the environment explained only a small proportion of the overall effect. These findings imply that cognitive processing of environmental conditions may play a limited role in behaviour change, and that high-quality changes to the physical environment itself—rather than changing people's perceptions of their environment—may be the key to the effectiveness of this type of intervention. Studies of how interventions lead to behaviour change should complement those concerned with estimating their effects in supporting valid causal inference in public health research.
